# Stimulation of the right dorsolateral prefrontal cortex reduces conflict-induced forgetting

**DOI:** 10.1093/cercor/bhaf279

**Published:** 2025-10-14

**Authors:** Oscar Kovacs, Carlo Miniussi, Olga-Lucia Gamboa, Nicolas A McNair, Irina M Harris

**Affiliations:** School of Psychology, University of Sydney, Brennan-MacCallum Building A18, Sydney, NSW 2006, Australia; Centre for Mind/Brain Sciences - CIMeC, University of Trento, Corso Bettini 31, 38068 Rovereto (TN), Italy; School of Psychology, University of Sydney, Brennan-MacCallum Building A18, Sydney, NSW 2006, Australia; School of Psychology, University of Sydney, Brennan-MacCallum Building A18, Sydney, NSW 2006, Australia; School of Psychology, University of Sydney, Brennan-MacCallum Building A18, Sydney, NSW 2006, Australia

**Keywords:** inhibitory control, memory retrieval, retrieval-induced forgetting, TMS

## Abstract

Retrieval-induced forgetting is a phenomenon whereby retrieving certain memories can impair the recall of related information. We tested the hypothesis that inhibitory mechanisms play a pivotal role in retrieval-induced forgetting, applying transcranial magnetic stimulation to the dorsolateral prefrontal cortex, an area involved in inhibitory control, while participants retrieved memory associations. Participants learned a series of word pairs, then completed an interference task where they learned directly conflicting associations, or semantically related associates. A train of transcranial magnetic stimulation pulses was applied to the right dorsolateral prefrontal cortex (Experiment 1) or the left dorsolateral prefrontal cortex (Experiment 2) during retrieval of the interfering pairs, beginning 200 ms after stimulus onset. Participants showed robust retrieval-induced forgetting for the original pairs after retrieving conflicting associations, which was reduced when transcranial magnetic stimulation was applied to the right dorsolateral prefrontal cortex compared with a control brain site. This effect was specific to conflicting memories and did not extend to semantically related interference, indicating the right dorsolateral prefrontal cortex’s critical role in inhibitory processes during retrieval. Transcranial magnetic stimulation administered to the homologous left dorsolateral prefrontal cortex had no effect on forgetting. These findings provide strong evidence for the involvement of the right dorsolateral prefrontal cortex in managing retrieval competition within an early time window (200 to 533 ms) and highlight its importance in memory control.

## Introduction

Retrieving memories usually strengthens the associated memory traces, though it can also reduce the accessibility of related information, a phenomenon known as retrieval-induced forgetting (RIF) (Anderson et al. 1994). In the most common RIF paradigm, participants study category–exemplar pairs of items (eg *Fruit-Apple, Fruit-Banana; Tool-Hammer, Tool-Scissors,* etc.), followed by a practice phase in which a subset of the items from a particular category (eg *Fruit*) are cued for retrieval (eg *Fruit-A___?*), and finally a test phase probing memory of all the originally studied items. Typically, items that have undergone retrieval practice (eg *Apple*) are remembered more accurately than control items from a nonretrieved category (eg *Tools*), while the unretrieved items from the practiced category (eg *Banana*) are recalled less accurately than control items, despite equivalent exposure. Much of the available evidence suggests that this RIF effect is the result of inhibition of items that compete with the target stimuli during retrieval (Storm and Levy 2012; Murayama et al. 2014), although an alternative account attributes it to competition within associative/semantic networks. According to the latter account, strengthening the retrieved information leads to a relative weakening of associated items, rendering them less accessible (Raaijmakers 2018). The use of related category–exemplar pairs in the typical RIF paradigm makes this competitive account more plausible, but a similar form of forgetting can be demonstrated using unrelated words, in an “interference” paradigm. Here, participants learn a set of stimulus associations (A–B pairs) and then must learn conflicting pairs of items (A–C). Retrieval practice of the new A–C pairs typically leads to poorer recall of the original A–B items, compared with items that do not receive interference from competing associations (ie no A–C item was practiced for that A–B pair). This interference paradigm has the advantage that it is easier to separate the contributions of inhibitory and associative processes, making it ideal for studying cognitive and neural processes involved in RIF.

Previous neuroimaging studies provide evidence for inhibitory mechanisms in RIF, by showing increased engagement of frontal lobe inhibitory mechanisms during memory retrieval, and a correlation between the strength of the frontal activation and the amount of later forgetting of competing information (Kuhl et al. 2007; Wimber et al. 2009). Across a range of memory inhibition paradigms, the area most consistently implicated is the right dorsolateral prefrontal cortex (DLPFC) (Anderson and Hanslmayr 2014; Anderson and Hulbert 2021). The underlying mechanism of retrieval inhibition is thought to involve signals from DLPFC projecting via the anterior cingulate cortex to the hippocampus, where they stimulate inhibitory GABA interneurons, thus reducing hippocampal activity during memory retrieval (Rizio and Dennis 2013; Anderson et al. 2016; Schmitz et al. 2017).

The present study aims to provide pivotal evidence for the right DLPFC in retrieval inhibition using online transcranial magnetic stimulation (TMS). TMS is a noninvasive brain stimulation technique with good spatial and temporal resolution that produces a brief perturbation in the neural activity at the site of stimulation. We used an A–B/A–C memory interference task and applied TMS to the right DLPFC (or control sites) during the retrieval practice of A–C pairs of items (ie the Interference phase). We reasoned that to successfully retrieve the new A–C item associations, the previously learnt conflicting A–B associations would need to be inhibited, resulting in poor recall of the original B item—the typical RIF effect. We hypothesized that if online TMS to the right DLPFC disrupts retrieval inhibition, we should see a reduction in RIF relative to when TMS is applied to control sites not involved in inhibition (the homologous contralateral DLPFC and the head Vertex/Cz). Furthermore, the effect of TMS to the right DLPFC should be specific to tasks that require retrieval inhibition of conflicting competitors and should not extend to other forms of interference, namely those caused by semantically related memories that do not directly compete for association with the cue.

## Materials and methods

### Participants

A total of 50 first-year students from the University of Sydney participated in exchange for course credit. Twenty-four took part in Experiment 1 (*M*_age_ = 19.17; 6 males and 18 females; 20 right-handed) and 26 took part in Experiment 2 (*M*_age_ = 20.96; 3 males and 23 females; 19 right-handed). All participants spoke English as their first language and did not report any risk factors for TMS on a safety screening questionnaire (Rossi et al. 2009).

### Ethics approval

The procedures were approved by the University of Sydney Human Research Ethics Committee (Protocol 2021-104) and all participants provided informed consent before commencing the study.

### Apparatus and stimuli

The experiment was programmed and run in PsychoPy version 2021.1 (Peirce 2007; Peirce et al. 2019; http://psychopy.org/). Participants viewed the display on a 26-inch LCD monitor with a resolution of 1,920 × 1,080 from a viewing distance of 70 cm.

The stimuli comprised 210 English words, 4 to 11 letters in length. Forty-eight pairs of unrelated words (denoted as A–B pairs; eg “Gallop-Winter”) were initially created for use in the Learning phase of the memory task. From each of these pairs, we then created 2 types of pairs to be used in the Interference phase of the memory task: a *Conflicting* pair (denoted as A–C pairs; eg “Gallop-Brush,” in which the original A cue stimulus was now paired with a different unrelated word) and an *Associated* pair (denoted as B_1_–B_2_ pairs; both words being close associates of the B word, eg “Autumn-Cold” for the B item “Winter”). We used Latent Semantic Analysis (LSA) to calculate word relatedness, with a minimum LSA cut-off of 0.6 between the B–B_1_ and B–B_2_ words.

### Memory task

The task consisted of 3 phases: Learning, Interference, and Final Recall Test. During the Learning phase, participants learned 48 word pairs (A–B pairs). To facilitate encoding, these were divided into 2 sets of 24, and each set was learnt to criterion as follows, before moving on to the next set. First, the word pairs were displayed on the screen and participants were free to look at them for as long as they liked, up to a maximum of 6 s, and then moved on to the next trial by pressing the space bar. If the participant did not respond within 6 s, the next trial began automatically. Participants were asked to try to form an association between the words and remember the pair. After seeing all 24 pairs in the set, they viewed the set again, in a different random order, this time with a fixed-time presentation of 3.5 s per item and a 0.75 s inter-trial interval. Following this second encoding attempt, participants’ memory was tested by presenting, in a different random order, the first word of each pair plus the first letter of the second word (eg Gallop—W__), which they had to retrieve and type in. Participants received feedback if their answer was incorrect, but were not told what the correct answer was. The encoding/retrieval practice cycle was repeated until they reached a criterion of 75% correct or for a maximum of 3 attempts.

**Fig. 1:**
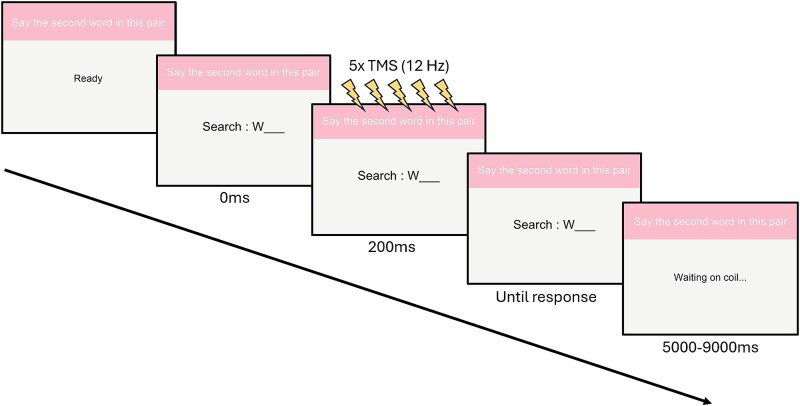
TMS trial structure. When each retrieval practice trial in the Interference phase began, participants were shown the first word from each pair and the first letter of the second for 200 ms before the onset of TMS stimulation. After stimulation, participants gave their response, then started a variable inter-trial interval of 5 to 9 s.

After the Learning phase was completed, participants moved to the Interference phase, in which they had to learn 32 new pairs of words. Half of these were *Conflicting* (A–C) pairs and half were *Associated* (B_1_–B_2_) pairs, derived from the originally learnt A–B pairs (see Stimuli). The remaining 16 A–B pairs from the Learning phase were not related to any stimuli in the Interference phase and constitute the Control (no interference) condition. The 32 word pairs were divided into 2 blocks of 16, which contained equal numbers of *Conflicting* and *Associated* pairs (8 each). TMS was administered during each block either to the DLPFC or the Vertex, with the order counterbalanced across participants. Each block followed the procedure of the Learning phase: participants first viewed all the word pairs at their own pace, then they were presented again with a fixed timing of 3.5 s per item and 0.75 s inter-trial interval. After this, they were presented with the first word of the pair (A or B_1_) along with the first letter of the second word (C or B_2_) and had to retrieve the second word and say it out loud. The order of the trials was randomized both between runs within participants and between participants. TMS was administered during these retrieval attempts (see below and Fig. 1). This encoding/retrieval practice sequence was repeated twice (unlike the initial Learning phase, where items were learnt to a criterion or maximum of 3 times, for this phase, we kept the number of learning and retrieval trials fixed to 2 in order to equate the amount of stimulation and interference across participants). Participants did not receive feedback on their responses in this phase.

In the Final Recall Test phase, participants were presented with the first word from each of the original A–B pairs in a random order (within each of the 2 sets) and asked to type in the second word of the original pair from the Learning phase. The first letter of the target word was not presented in this phase, and no feedback was given. Recall accuracy in this final test was restricted to the items that participants had successfully recalled during the Learning phase, and was analyzed as a function of the type of interference that each tested pair had received during the Interference phase—ie *Conflicting* or *Associated* interference or no interference (*Control*).

### Transcranial magnetic stimulation procedure

After a brief introduction to TMS and the completion of a safety screening questionnaire (Rossi et al. 2009), participants were prepared for TMS. TMS pulses were administered using a Magstim Rapid^2^ with a D70^2^ 70 mm figure-eight coil (Magstim, Whitland, United Kingdom). Electromyogram was recorded with bipolar montage: 2 Ag/AgCl surface electrodes were attached in a belly-tendon configuration over the participant’s right first dorsal interosseous (FDI) muscle, with a ground electrode placed on the ulnar styloid process of the wrist. Motor evoked potentials (MEPs) were measured using a PowerLab 26 T data acquisition device (ADInstruments, Sydney, Australia) and recorded for offline analysis with LabChart version 8 software. Motor thresholds were estimated using the staircasing method implemented in the ATH-tool app (v. 2020 August 27; Julkunen 2019), running on an Android tablet. The participant’s resting motor threshold (rMT) was used as the basis for the intensity of stimulation during the subsequent experimental task.

Participants wore a Lycra cap with the 10 to 20 electroencephalography (EEG) electrode positions marked on it. The experimenter first positioned the coil over the left primary motor cortex (M1) in a position tangential to the scalp, and angled ~45° away from the interhemispheric fissure, with the handle pointing backwards. The location of the motor hotspot was estimated by starting from a location 5 cm lateral and 1 cm anterior to the Cz/Vertex electrode location and moving the coil until a maximal MEP was elicited in the FDI muscle. When the optimal location was found, the participant’s head was restrained with the aid of a chin rest and the coil was locked in place using a variable friction mechanical arm (244 N; Manfrotto, Cassola, Italy). The participant’s rMT was defined as the minimal stimulator output required to elicit a 50 μV peak-to-peak amplitude MEP, determined using the staircasing procedure implemented in the ATH-tool. The ATH tool provides an estimate of the rMT based on 20 staircase trials until a 5% CI has been reached. The stimulation intensity during the experiment was set to 110% of rMT, which resulted in a mean of 46.2% (SD = 7.5%) of the maximum stimulator output in Experiment 1 and 40.4% (SD = 10.2%) in Experiment 2.

The stimulation sites were determined by co-registering each participant’s head space mapped from fiducial points to the MNI template, using Brainsight 2.4 neuronavigation software (Rogue Research, Montreal, Canada) with a Polaris Vicra tracker system (Northern Digital, Waterloo, Canada). The nasion, left preauricular, and right preauricular points were first digitized on the participant’s head using the tracking system and were used to align the corresponding locations within the Montreal Neurological Institute (MNI) template to the participant’s fiducial points. The outermost parts of the participant’s scalp (top, back, sides, and forehead) were then digitized and used to warp the MNI template to fit the participant’s headspace. Co-registration was confirmed by an error <5 mm between the final warped template and majority of these points on the participant’s head; otherwise, the procedure was redone until a close match was obtained. The resulting co-registered MNI template was used to visualize the location of the targets and guide the positioning of the coil.

The experimental TMS sites were the right (Experiment 1) and left (Experiment 2) DLPFC, corresponding to Talairach coordinates: *x* = ±40, *y* = 32, and *z* = 30. These coordinates were based on TMS studies looking at the modulation of DLPFC connectivity in multimodal interference tasks (Paus et al. 2001; Strafella et al. 2001; Cohen and Robertson 2011). The control site was the Vertex, corresponding to Talairach coordinates: *x* = 0, *y* = 11, and *z* = 69 (Okamoto et al. 2004). These Talairach coordinates were used to set the stimulation targets in the neuronavigation software, which converted these to MNI coordinates. For the DLPFC stimulation, the coil was positioned over the respective site with the handle angled at 45° from the midline fissure and pointing up. In the Vertex stimulation, the coil was positioned over Cz, with the handle pointing backward toward the occipital cortex. The coil was locked into position with the aid of a mechanical arm and neuronavigation software was used to ensure that it remained within 1 mm of the stimulation target. TMS preparation was conducted prior to the Learning phase, but the participant’s head was not secured until just prior to the commencement of the Interference phase.

TMS was delivered during the retrieval practice trials of the Interference phase of the experiment (ie online TMS)—ie when the participants were cued with the first word of the pair and had to produce the second word. On each retrieval practice trial, a train of 5 pulses were delivered with a frequency of 12 Hz (333 ms total duration; following Harris et al. 2008) starting 200 ms after presentation of the cue word (ie covering a time window 200 to 533 ms post cue onset). The inter-trial interval was jittered between 5 and 9 s, to prevent any potential build-up effects of TMS and reduce expectancy effects (Vaseghi et al. 2015; Pellicciari et al. 2016), see Fig. 1. The location of stimulation was manipulated within-subjects, with each participant receiving TMS to the DLPFC and the Vertex locations, as they performed blocks 1 and 2 of the Interference phase, with the order of the blocks counterbalanced across participants. All participants completed 2 retrieval practice cycles for each word pair per block, resulting in a total of 32 TMS trials for each stimulation location. To minimize movement, participants gave their responses aloud rather than typing them in and responses were recorded using Audacity software (The Audacity Team, Pittsburgh, USA) and transcribed after the experimental session was over.

## Experimental design and analysis

Our primary measure of interest was the proportion correct recall on the final test. In both experiments, we used Bayesian repeated-measures analysis of variance (ANOVA), using the Bayes analysis module in JASP version 0.19.1. Bayesian analyses are preferable to frequentist statistics, as they provide evidence for the null and alternative hypotheses (Rouder et al. 2009; Dienes 2016). For all analyses, we used the default priors in JASP (a Cauchy distribution centered on 0 with a width parameter of √2/2). Following accepted conventions, a Bayes factor (BF) > 10 was considered strong evidence in favor of the alternative hypothesis and BF > 3 was considered moderate evidence in favor of the alternative hypothesis. On the other hand, 0.1 < BF < 0.33 was considered moderate evidence in favor of the null and BF < 0.1 was considered strong evidence in favor of the null. Values in between indicate insufficient evidence for either hypothesis.

First, we assessed the effects of interference in the Vertex (control) condition by comparing accuracy for the *Conflicting* and *Associated* items to *Control* items. In both experiments, we expected to find stronger evidence for interference—and consequently RIF—in the *Conflicting* compared with the *Associated* condition. Next, in each experiment, we used a 2 × 2 ANOVA with the factors of Interference Type (*Conflicting* vs *Associated*) and Stimulation Site (*DLPFC* vs *Vertex*) to assess the effects of DLPFC stimulation on the amount of forgetting induced by each form of interference. We expected to find evidence of an interaction in Experiment 1, with stimulation of the right DLPFC reducing the amount of RIF suffered by the *Conflicting* items compared with stimulation of the Vertex, and no effect of TMS on the RIF suffered by the *Associated* items. We did not expect to find such an interaction in Experiment 2, given that the corresponding DLPFC location in the left hemisphere is not thought to play a role in memory inhibition. The JASP analysis files are available at https://osf.io/4fthx/.

Secondary analyses using the same 2 × 2 ANOVA were also run on the data from the final interference trial to measure the effects of interference type and TMS site on the learning of the alternative targets associated with the retrieval cue.

Finally, to compare across both experiments and determine whether there was a differential effect of TMS to the right vs left DLPFC, we used the region of practical equivalence test (ROPE; Kruschke 2015, 2018), following the “full ROPE” approach recommended by Kelter (2020). This analysis was run in R, using the brms and bayestestR packages (Makowski et al. 2019; Bürkner 2021; Posit team. 2025) to derive full posterior distributions of the Interference Type by Stimulation Site interactions from each experiment, based on the default JASP priors. The difference between these distributions was compared to a [−0.1^*^SD, 0.1^*^SD] ROPE. Lower percentages of the posterior within the ROPE indicate stronger evidence for an effect, with values <2.5% indicating that the null should be rejected, and values >97.5% indicating that the null should be accepted (Makowski et al. 2019). The R script is available at https://osf.io/4fthx/.

## Results

### Experiment 1

#### Performance during the Learning and Interference phases

During the Learning phase, participants had 3 attempts to reach a minimum accuracy of 0.75 for each set of 24 items, which each participant did successfully, taking an average of 1.29 (SD *=* 0.54) attempts per set. Mean accuracy for the first attempt that passed the minimum threshold was 0.895 (SD = 0.307). In the Interference phase, participants had 2 retrieval attempts and achieved a mean accuracy of 0.983 (SD = 0.131) on the second and final attempt, indicating very robust learning of the interference materials (see Table 1 for accuracy across conditions). A Bayesian ANOVA performed on the accuracy of the final interference trial, using Interference Type (*Associated* vs *Conflicting*) and TMS location (*right DLPFC* vs *Vertex*) as within-subject factors did not find any evidence of either effect during the Interference phase, ie when TMS was actually being delivered (BF_incl_ = 0.653 and BF_incl_ = 0.314, for the interference and TMS site effects, respectively). Importantly, these results can be considered as moderate evidence in favor of a null effect of TMS on the learning of the conflicting (new) material, although the evidence for an effect of type of interference is inconclusive.

**Table 1:** Accuracy on the final test of the Interference phase.

	**Associated** **vertex**	**Associated** **DLPFC**	**Conflicting** **vertex**	**Conflicting** **DLPFC**
Experiment 1	0.99 (0.07)	0.99 (0.10)	0.98 (0.14)	0.97 (0.17)
Experiment 2	0.99 (0.12)	0.98 (0.15)	0.93 (0.25)	0.92 (0.27)

#### Performance during the Final Recall Test

We removed data on the final test trials for any word pairs that had not been learned successfully during either the Learning or Interference phases (12% of 1,152 total trials). Overall proportion correct averaged 0.679 (SD = 0.142) across conditions. A preliminary analysis indicated that accuracy on the final test did not correlate with the success in learning the new associations during the interference test for any of the conditions (Pearson’s correlation coefficients *r*s < 0.11, *p*s > 0.14), suggesting that any effects observed on the final test are not due to learning the new associations, but rather to RIF.

**Fig. 2:**
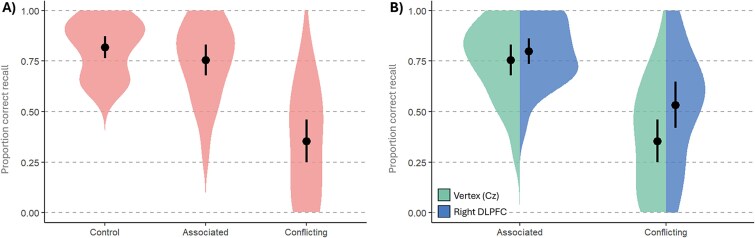
Experiment 1 Final Recall Test accuracy. (A) Proportion correct accuracy in the Vertex TMS condition, with mean performance denoted by the point, and a 95% CI indicated by the black bar. (B) Proportion correct accuracy as a function of interference type and TMS site, with mean performance denoted by the point, and a 95% CI indicated by the black bar.

To establish the effects of interference on final recall accuracy, we compared final recall accuracy of the *Control* (no interference) items (*M* = 0.815, SD = 0.136) and the *Conflicting* (*M* = 0.357, SD = 0.258) and *Associated* (*M* = 0.760, SD = 0.191) items for the block of trials that received TMS to the Vertex (see Fig. 2A). There was very strong evidence in favor of the alternative hypothesis that performance differed between conditions (BF_10_ = 1.532 × 10^12^). There was very strong evidence for interference in the *Conflicting* condition (BF_10_ = 1.478 × 10^7^) and inconclusive evidence for an interference effect in the *Associated* condition (BF_10_ = 0.937).

To assess the effect of TMS to the DLPFC during the retrieval practice of interfering items, we conducted a Bayesian ANOVA with Interference Type (*Conflicting* vs *Associated* items) and TMS Site (*right DLPFC* vs *Vertex*) as within-subject factors. This analysis provided strong evidence for a main effect of Interference Type (BF_incl_ = 6.726 × 10^5^), moderate evidence for a main effect of TMS Site (BF_incl_ = 3.917), and moderate evidence for a TMS Site × Interference Type interaction (BF_incl_ = 5.488). Specifically, TMS to the right DLPFC reduced the interference suffered by the *Conflicting* items (*m*_diff_ = 0.176) to a greater degree than the Associated items (*m*_diff_ = 0.025), see Fig. 2B. The data are available at https://osf.io/4fthx/.

### Experiment 2

#### Performance during the Learning and Interference phases

During the Learning phase, participants took an average of 1.46 (SD *=* 0.73) attempts to learn each set of items to the 0.75 accuracy criterion. Mean accuracy for the first attempt, which passed this minimum threshold, was 0.921 (SD = 0.275), slightly higher than in Experiment 1. In the Interference phase, average accuracy was 0.961 (SD = 0.210) for the second of the 2 retrieval practice attempts (see Table 1). A Bayesian ANOVA performed on the accuracy of the final interference trial, using Interference Type (*Associated* vs *Conflicting*) and TMS location (*left DLPFC* vs *Vertex*) as within-subject factors revealed moderate evidence of a null effect of TMS on the learning of the interfering material (BF_incl_ = 0.260), and inconclusive evidence of an effect of interference type (BF_incl_ = 1.094), consistent with the findings of Experiment 1.

#### Performance on the Final Recall Test

Trials containing any items that were not successfully recalled at the end of the Learning phase, or during the Interference phase, were removed from the final recall analysis (10% of 1,248 total trials). Mean recall accuracy across conditions was 0.711 (SD = 0.137), which is slightly higher than in Experiment 1. As in Experiment 1, a preliminary analysis indicated that accuracy on the final test did not correlate with the success in learning the new associations during the interference test for any of the conditions (Pearson’s correlation coefficients −0.14 < *r*s < 0.07, *p*s > 0.35), suggesting that learning the new associations was not the main driver of the effects observed on the final test.

**Fig. 3:**
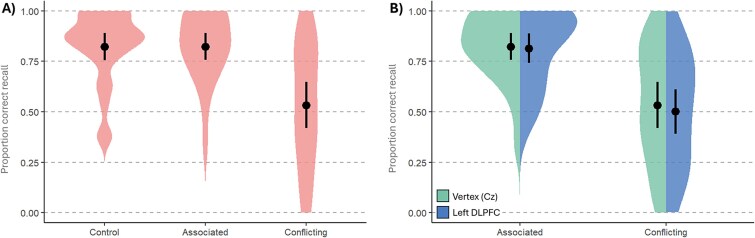
Experiment 2 Final Recall Test accuracy. (A) Proportion correct accuracy in the Vertex TMS condition, with mean performance denoted by the point, and a 95% CI indicated by the black bar. (B) Proportion correct accuracy according to interference type and TMS site, with mean performance denoted by the point, and a 95% CI indicated by the black bar.

Similar to Experiment 1, we first established whether there were interference effects on final recall by comparing accuracy for the *Control* items (*M* = 0.815, SD = 0.189) with accuracy for items that had received *Conflicting* interference (*M* = 0.528, SD = 0.249) or *Associated* interference (*M* = 0.813, SD = 0.177) during TMS to the Vertex (see Fig. 3A). As in Experiment 1, there was very strong evidence in favor of the alternative hypothesis that performance differed between conditions (BF_10_ = 9.391 × 10^5^). There was very strong evidence for interference in the *Conflicting* condition (BF_10_ = 4.718 × 10^4^) and moderate evidence against an interference effect in the *Associated* condition (BF_10_ = 0.207).

As in Experiment 1, the effects of TMS to the DLPFC during the retrieval practice of interfering items were assessed with a Bayesian ANOVA of the final recall performance, using TMS (*left DLPFC* vs *Vertex*) and Interference Type (*Conflicting* vs *Associated* items) as within-subject factors. This analysis provided strong evidence for a main effect of Interference Type (BF_incl_ = 2.695 × 10^4^). In contrast to Experiment 1, there was moderate evidence against a main effect of TMS Site (BF_incl_ = 0.243) and moderate evidence against the interaction between Interference Type and TMS Site (BF_incl_ = 0.223), indicating that TMS to the left DLPFC did not modulate the amount of RIF suffered by the original word pairs (see Fig. 3B). The data are available at https://osf.io/4fthx/.

### Experiments 1 and 2

To get a clearer picture of whether the interaction effect depended on the lateralization of DLPFC stimulation, the results of both experiments were directly compared using the full ROPE procedure (Kruschke 2015, 2018; Kelter 2020). We began by calculating the posterior distribution of the difference between the interaction effects in each experiment (diff_median_ = 0.27, 95% credible interval [−0.69, 1.23], pd = 70.43%), then compared this to the [−0.1^*^SD, 0.1^*^SD] ROPE. This posterior distribution was found to have only 2.12% overlap with the ROPE, indicating that the interaction effect did meaningfully differ across experiments, though based on the wide credible interval and relatively low directional certainty, this effect is likely to be quite variable. This result is consistent with the evidence for an interaction between Interference Type and Stimulation Site in Experiment 1 and evidence against the interaction in Experiment 2, suggesting that the interaction depends on the lateralization of DLPFC stimulation.

## Discussion

This study tested the involvement of inhibitory processes in RIF by disrupting the pattern of neural activity in a brain area—the right DLPFC—that is thought to play a significant role in inhibition across a range of cognitive and motor tasks. We used an interference task in which word cues were associated with multiple targets, and thus likely to compete with each other during retrieval (the conditions that typically result in RIF). Subjects learnt some initial associations (A–B) and then learnt conflicting (A–C) word pairs, with the expectation that retrieval practice of the A–C pairs would elicit inhibition of the original cue-target association, making it more difficult to recall these items during a later memory test, compared to items that had not received interference. As expected, we found robust RIF (ie forgetting of the original B targets) in both of our experiments. In Experiment 1, TMS delivered during retrieval practice of the A–C pairs, in a time window 200 to 533 ms after the onset of the cue, resulted in better recall of the original B items in a final test, compared with when TMS was applied to a control site (Vertex). This reduction in RIF provides evidence that the right DLPFC is critical for managing retrieval of competing memories. In contrast, when TMS was delivered to the homologous site in the left DLPFC in Experiment 2, there was no reduction in RIF compared with the Vertex. Given that the left DLPFC stimulation equates the sensory and distracting effects of TMS (eg uncomfortable facial twitches), we can be confident that the TMS effect seen here is not due to nonspecific effects and is more likely to be due to changes in cognitive processing.

Crucially, our results indicate that the right DLPFC plays a specific role in inhibitory processes that are used when 2 memories directly compete for association with a cue, as in our A–B/A–C *Conflicting* condition, rather than contributing to RIF that might be induced through other means. Some theories of RIF attribute the phenomenon to a relative decrease in item strength caused by associative competition, rather than active inhibition (for an overview, see Raaijmakers 2018). To address this possibility, our experiments also included a condition intended to create a high level of associative interference for the memory targets, by having participants initially learn A–B word pairs and then, during the interference phase, being exposed to close semantic associates of the target word (B_1_ and B_2_; eg “snow” and “autumn” for the B target “winter”). We found that TMS to the right (or, indeed, left) DLPFC did not affect performance in this condition, in direct contrast to its effect on RIF in the *Conflicting* condition. However, it is important to acknowledge that across both experiments, there was no evidence of forgetting in the *Associated* condition, so it is possible that the lack of a TMS effect is due to a ceiling effect. Note that our experiments deliberately separated the inhibitory and associative influences that could lead to forgetting, which tend to be conflated in most RIF experiments that use category labels as cues and exemplars of that category as competing targets. The results indicate that RIF is largely caused by inhibitory control processes used to deal with conflicting memories, with associative interference playing a minor role, if any.

The present findings are consistent with the results of an earlier study that used transcranial direct current stimulation (tDCS) applied over the right DLPFC during a standard retrieval practice paradigm and found a reduction in RIF after cathodal stimulation (Penolazzi et al. 2014). Our study has 2 advantages over this earlier study and extends it in important ways. First, tDCS is a relatively blunt method of stimulation, which was applied over a period of ~20 min during the entire retrieval practice phase of the experiment. In contrast, our study provides the first evidence that stimulation during a very specific time window (200 to 533 ms after the onset of the cue) is sufficient to reduce retrieval inhibition and consequent RIF. Second, as outlined above, our experiment was better able to isolate inhibitory processes from associative interference, so we can be more confident that the modulation of RIF in our experiment is due to a reduction in inhibition. And finally, our study also included a left DLPFC stimulation site, which helped to confirm a special role for the right DLPFC in inhibition of mnemonic competitors. In a further tDCS study, Stramaccia et al. (2017) also found some evidence for the involvement of the right inferior frontal gyrus (IFG) in memory inhibition and RIF. In their study, the standard RIF effect was only observed for some categories of stimuli, and its modulation by IFG stimulation was overall less pronounced. The authors attributed the weaker effects to the stimulus characteristics, although we cannot rule out the possibility that the IFG may not play as critical a role in memory control. This could be tested in future experiments using our interference paradigm, in which TMS is applied over the IFG.

Our online TMS protocol allowed us to selectively target a specific time window during the retrieval attempt. The stimulation (5 pulses with 12 Hz frequency) started 200 ms after the cue onset and lasted for 333 ms. We chose this time window based on EEG studies of RIF, which had identified frontally generated event-related potential (ERP) components in an early time window (200 to 400 ms after cue onset) during retrieval practice that were predictive of the amount of subsequent forgetting (Johansson et al. 2007; Hanslmayr et al. 2010). Our results provide causal evidence that right DLPFC activity at this time is indeed responsible for later RIF. This relatively rapid engagement of the right DLPFC is interesting given that the shift from sensory processing to memory retrieval itself is thought to start around 300 to 500 ms after stimulus onset (Friedman and Johnson 2000; Rugg and Curran 2007; Waldhauser et al. 2016; Staresina and Wimber 2019), meaning that there is little time for items to be retrieved and consciously evaluated before initiating DLPFC-dependent inhibitory processes. This suggests that frontal mechanisms of memory control are activated in tandem with identification of the cue and likely play a role in monitoring the retrieval process itself and inhibiting undesired memory associations that might interfere with current task goals.

A further important finding of these experiments is that TMS did not disrupt recall of the targets of the retrieval practice (the A–C pairs) even though it was administered during this recall. It is possible that we would have found an effect on recall of the practiced item had we stimulated in a later time window, as suggested by an EEG study (Hellerstedt and Johansson 2014), which found that activity in anterior regions 1,200 to 1,500 ms after the onset of the cue was significantly associated with retrieval practice success but was not predictive of subsequent RIF. However, it is worth noting that the tDCS studies described above (Penolazzi et al. 2014; Stramaccia et al. 2017) similarly did not find an effect of stimulation on the facilitated recall of the practiced items despite stimulating throughout the whole retrieval practice phase of the experiment. Taken together, these findings demonstrate that the right DLPFC plays a specific role in the monitoring and inhibition of competing memories, rather than in memory retrieval in general.

## Conclusion

The present study revealed a critical role for the right DLPFC in inhibiting conflicting memories during retrieval, a process that ordinarily leads to RIF. Our experiments singled out activity in the right DLPFC in an early time window (200 to 533 ms from encountering a cue) as a necessary step in selecting the appropriate target of memory retrieval to meet current task goals.
